# Assessing CD8^+^ T cell cross‐reactivity to influenza A, B and C virus‐derived peptides presented by HLA‐B*18:01

**DOI:** 10.1002/cti2.70123

**Published:** 2026-10-13

**Authors:** Lawton D Murdolo, Samuel Liwei Leong, Janesha C Maddumage, Lloyd D'Orsogna, Demetra SM Chatzileontiadou, Emma J Grant, Stephanie Gras

**Affiliations:** ^1^ Infection and Immunity Program La Trobe Institute for Molecular Science (LIMS) Bundoora VIC Australia; ^2^ Department of Biochemistry and Chemistry La Trobe University Bundoora VIC Australia; ^3^ School of Medicine University of Western Australia Nedlands WA Australia; ^4^ Department of Clinical Immunology and PathWest Fiona Stanley Hospital Murdoch WA Australia; ^5^ Department of Biochemistry and Molecular Biology Monash University Clayton VIC Australia

**Keywords:** CD8^+^ T cells, HLA‐B*18:01, influenza virus, T cell cross‐reactivity, X‐ray crystallography

## Abstract

**Objectives:**

Despite the availability of vaccines, influenza viruses still cause substantial morbidity and mortality. This highlights the need for improved vaccines and novel therapeutics. CD8^+^ T cells recognise conserved viral epitopes and can provide broad protection, making them attractive targets for next‐generation vaccine design. A key step in this process is to identify T cell epitopes across influenza viruses. Here, we characterised three homologous influenza Polymerase Basic 1 (PB1)‐derived peptides from influenza viruses (PB1‐A_310_, PB1‐B_310_ and PB1‐C_312_, respectively) presented by the common, but understudied, HLA‐B*18:01 molecule.

**Methods:**

We used a thermal melt assay and X‐ray crystallography to assess the stability and structural features of each peptide. CD8^+^ T‐cell responses were evaluated using intracellular cytokine staining with HLA‐B*18:01^+^ samples.

**Results:**

The structures of HLA‐B*18:01 presenting each peptide were solved, including the first structure of HLA‐B*18:01 presenting a *Gammainfluenzavirus* (formerly influenza C virus) derived peptide. PB1‐C_312_ exhibited a higher thermal stability and a different conformation compared to PB1‐A_310_ and PB1‐B_310_. CD8^+^ T cell responses varied among samples, with PB1‐A_310_ eliciting the strongest activation. Minimal T cell cross‐reactivity was observed and was mostly between PB1‐A_310_ and PB1‐B_310_ likely reflecting shared structural features.

**Conclusion:**

These findings further expand knowledge on peptide presentation by the common HLA‐B*18:01 molecule, and the limitations on CD8^+^ T cell cross‐recognition of this PB1‐derived epitope across influenza lineages. This highlights the importance of more research on cross‐lineage T cell cross‐reactivity that could inform the design of broadly protective T cell‐based vaccines and therapeutics.

## Introduction

Respiratory tract infections caused by influenza viruses represent a significant public health concern, costing the United States of America (USA) healthcare system alone an estimated $6.3 to $25.3 billion annually.^1^ Of the four known influenza virus types, three are associated with human infections: *Alphainfluenzavirus influenzae* (FLUAV, formerly influenza A virus), *Betainfluenzavirus influenzae* (FLUBV, formerly influenza B virus) and *Gammainfluenzavirus influenzae* (FLUCV, formerly influenza C virus).^2^ Among these, FLUAV is the most notable, having caused four pandemics since the early 20th century.^3^ The most recent 2009 swine influenza pandemic resulted in an estimated 105 700 to 395 600 deaths within the first 12 months of the outbreak.^4^ While FLUBV is considered less infectious, it still contributes to a considerable disease burden, accounting for approximately 23% of all influenza cases in humans each season, although this can vary substantially between years.^5^ Notably, children (< 18 years) are particularly vulnerable, with FLUBV responsible for 16–52% of all influenza associated paediatric deaths in the United States each season.^6^ FLUCV is generally associated with mild illness in adults and considered the least severe of the three. However, infections are likely highly underreported because of the difficulty in isolating the virus within cell cultures.^7^ Despite this, serological studies from the 1980s indicate that in some populations, up to 90% of children aged 7–10 years show evidence of previous infection with FLUCV.^8^ In addition, FLUCV can trigger severe respiratory illnesses in children < 2 years of age.^9^ Overall, influenza viruses continue to pose a major global health threat, with approximately 3–5 million cases and 290 000–650 000 deaths worldwide.^10^ It is therefore crucial to improve current treatments to combat these viruses.

The majority of current influenza vaccines prime the humoral adaptive immune response leading to the production of neutralising antibodies predominately against the influenza virus surface glycoprotein called haemagglutinin (HA) from multiple influenza virus strains, thereby preventing viral entry into host cells.^11^ The effectiveness of these vaccines depends on several factors. First is age, with clinical evidence indicating that individuals over the age of 65 or under the age of 18 respond better to certain formulations.^12^, ^13^, ^14^, ^15^ Second, circulating strains of FLUAV and FLUBV frequently undergo antigenic drift, resulting in gradual accumulations of mutations,^16^ and FLUAV possesses the added capacity for antigenic shift, a rapid reassortment of gene segments^17^ because of the presence of viral reservoirs in animals. Mutations within the HA surface glycoprotein can reduce antibody binding and vaccine efficacy by approximately 10–60%,^18^ and have the potential to result in the emergence of novel viral subtypes in populations lacking prior immunity. Therefore, to keep up with the changes in circulating influenza strains, vaccines must be re‐formulated to target predicted predominant circulating strains, up to 6 months in advance, at a cost of $68 billion annually.^19^ Furthermore, if these predictions are inaccurate, vaccine effectiveness can drop significantly, with reports of reductions to as low as 19% in the United States.^20^ Given the substantial global health threat posed by influenza, the development of a novel and effective vaccine and therapeutic strategies remain a critical priority.

It has been well‐established that CD8^+^ T lymphocytes (CD8^+^ T cells) provide protection against influenza infection through cytokine production and directly killing virus infected cells, which reduces the severity of disease.^21^, ^22^ This can also be achieved independently of neutralising antibodies.^23^, ^24^, ^25^ CD8^+^ T cells could provide broad protection and have been shown to recognise conserved internal influenza proteins^26^, ^27^ that are generally less susceptible to mutation.^28^ The activation of CD8^+^ T cells requires their surface bound T‐cell receptor (TCR) to recognise different pathogenic peptides (epitopes) presented by the Human Leukocyte Antigens class I (HLA‐I) molecules. HLA molecules are present on the surface of all nucleated cells,^29^ including pathogen infected cells. There are over 29 000 *HLA‐I* alleles currently identified.^30^ Each allomorph contains a unique binding cleft composed of two domains (α1 and α2) housing six biochemically distinct binding pockets (A–F).^31^ Each HLA presents a specific set of peptides, mainly determined by the biochemical nature of its B pocket that bind to the second peptide residue (P2) and its F pocket that bind to the last peptide residue (PΩ).^31^

There are a vast number of potential peptides that could be presented by HLA molecules but the number of CD8^+^ T cells within an individual is finite. In most cases, each CD8^+^ T cell expresses a single TCR, with an estimated 10^7^–10^8^ distinct T cell clonotypes present in each individual.^32^ Consequently, a single TCR must be capable of recognising more than one peptide presented by an HLA (peptide‐HLA, or pHLA), a phenomenon known as T cell cross‐reactivity^33^ that could provide broad immune protection. Once activated, T cells can form a memory CD8^+^ T cell pool that can recognise and respond to rapidly mutating RNA viruses such as influenza.^34^ Understanding the features of epitopes that can stimulate cross‐reactive CD8^+^ T cells is critical for the rational design of vaccines capable of inducing broad and durable protection against multiple viral strains.^25^ Therefore, to identify potential T cell epitopes that could be used in T cell‐based therapeutics, it is essential to gain a deeper understanding of peptide presentation by different HLA‐I molecules.

HLA‐B*18:01 is the 15th most expressed *HLA‐B* allele in humans and present in ~2% of the world's population,^35^ reaching population frequencies of >15% in central Europe and > 20% in south east Asia.^36^ Despite this relatively high frequency in theses populations, the HLA‐B*18:01 molecule remains relatively understudied in the context of influenza infection, with only five influenza‐derived peptides structurally characterised. Of these five influenza peptides, four are FLUAV‐derived, including the immunodominant Nucleoprotein (NP) derived NP_219_,^37^ and one is FLUBV Polymerase Basic 1 (PB1)‐derived, PB1_177‐B_.^38^ There is limited information on FLUCV‐derived epitopes and none are reportedly HLA‐B*18:01‐restricted.^39^ This highlights a gap in our understanding of the immune response to FLUCV, a virus that can causes serious illness in children.

Here, we present a structural and immunological analysis of three homologous peptides derived from the internal PB1 protein across FLUAV (^310^NENQNPRMF^318^, PB1‐A_310_), FLUBV (^310^NECLNPRIF^318^, PB1‐B_310_) and FLUCV (^312^NECQQPEAY^320^, PB1‐C_312_). All three peptides contain the favored HLA‐B*18:01 binding anchor residues at Position 2 (P2‐Glu) and Position 9 (P9‐Phe/Tyr).^40^ Crystal structures of the pHLA complexes reveal that HLA‐B*18:01 presents PB1‐A_310_ and PB1‐B_310_ in a similar conformation but that the PB1‐C_312_ conformation is different and associated with an increased stability. The activation and cross‐reactivity of CD8^+^ T cells in HLA‐B*18:01^+^ samples were assessed. The data show that PB1‐A_310_ was the most immunogenic peptide, followed by PB1‐B_310_, while limited T cell activation was observed towards the PB1‐C_312_ peptide. The level of CD8^+^ T cell cross‐reactivity observed was minimal, suggesting that these PB1‐derived epitopes might not confer broad cross‐protective immunity against different influenza strains.

This study provides insights into peptide presentation by a common HLA‐I molecule and highlights the constraints of T‐cell cross‐recognition across different influenza strains. This could help to inform future CD8^+^ T cell‐based therapeutics and vaccines, aimed at improving cross‐strain protection.

## Results

### PB1‐A_310_ and PB1‐B_310_ exhibit a different conformation and lower stability than the PB1‐C_312_ peptide

We first assessed the stability of each peptide‐HLA‐B*18:01 complex. The HLA‐B*18:01 in complex with the PB1‐A_310_ and PB1‐B_310_ peptides exhibited similar thermal stability (*T*_m_ ~49°C) (Table 1, Supplementary figure 1). The *T*_m_ values were 10°C lower than those reported for other immunogenic HLA‐B*18:01‐restricted epitopes,^37^, ^41^, ^42^ but comparable to a published non‐immunogenic FLUBV peptide PB1‐B_177_ (Table 1).^38^ In contrast, PB1‐C_312_ exhibited a 14°C higher melting temperature than PB1‐A_310_ and PB1‐B_310_, comparable to other immunogenic HLA‐B*18:01 peptides (Table 1, Supplementary figure 1).

**Table 1:** Peptide‐HLA‐B*18:01 complex characteristics

Peptide	Peptide sequence	*T*_m_ (°C) ± SEM	PDB code	Secondary anchor residue
PB1_177‐B_	PEMTFFSVK	48.92 ± 1.25	9DY8^38^	P5‐Phe
PB1‐A_310_	NENQNPRMF	49.02 ± 0.08	9ZZ7 (this study)	P7‐Arg
PB1‐B_310_	NECLNPRIF	49.90 ± 0.07	9ZZ8 (this study)	P7‐Arg
NP_338_	FEDLRVSF	55.70 ± 1.20	6MT3^43^	P5Arg
NS2_111_	QEIRTFSF	61.14 ± 0.72	8ROP^37^	P5‐Thr
M1_5_	TEVETYVL	62.51 ± 0.04	8RNG^37^	P5‐Thr
NP_219_	YERMCNIL	63.00 ± 0.08	8ROO^37^	P5‐Cys
PB1‐C_312_	NECQQPEAY	63.99 ± 0.18	9ZZN (this study)	P5‐Gln
PB1_177‐A_	EEIEITTHF	64.45 ± 0.99	8RNH^37^	P5‐Ile
BZLF1	SELEIKRY	68.80 ± 0.30	4JQV^42^	P5‐Ile
Self	DELEIKAY	72.30 ± 1.30	4XXC^41^	P5‐Ile

In this study, the thermal stability experiments were performed at two concentrations (5 and 10 μm), in duplicate, and with two independent replicates (*n* = 2). Hydrogen bond interaction defined with superscript and within ≤ 3.5 Å, determined in PyMoL.

SEM, standard error of the mean; *T*_m_, thermal midpoint temperature.

We then solved the crystal structures of each peptide presented by HLA‐B*18:01 at high resolution (Supplementary table 1), with all three peptides able to be presented by HLA‐B*18:01 (Figure 1). A well‐defined electron density map was obtained for each peptide and revealed a canonical extended orientation in the HLA binding cleft (Figure 1a–c, Supplementary figure 2). The primary anchor P2‐Glu formed a hydrogen bond with the His9 in the B pocket of HLA‐B*18:01 for all peptides (Supplementary figure 3a), similarly to all published HLA‐B*18:01 structures.^37^, ^38^, ^41^, ^42^ The side chains of the P9 residues of each peptide bind the F pocket through a network of hydrophobic interactions (Supplementary table 2). The HLA‐B*18:01‐PB1‐C_312_ structure shows that the hydroxyl group of P9‐Tyr forms a hydrogen bond with Ser116 at the base of the cleft (Supplementary figure 3b), a conserved interaction observed in other HLA‐B*18:01 structures containing peptides with a PΩ‐Tyr residue.^41^, ^42^ Interestingly, both PB1‐C_312_ and the previously published peptides with a P9‐Tyr, all exhibited a high *T*_m_ in complex with HLA‐B*18:01 (Table 1). PB1‐A_310_ and PB1‐B_310_ presented by HLA‐B*18:01 both adopt a similar conformation, with a peptide root‐mean‐square deviation (r.m.s.d.) of 0.183 Å following alignment of the binding clefts (Figure 1d). In both complexes, the side chains of P4‐Gln/Leu, P5‐Asn, P6‐Pro and P8‐Met residues were solvent exposed and represent potential contacts for a TCR (Figure 1a and b). Furthermore, P7‐Arg for both peptides acted as a secondary anchor, forming a salt bridge with Asp114 within the D pocket of the HLA (Figure 2a and b).

**Figure 1:**
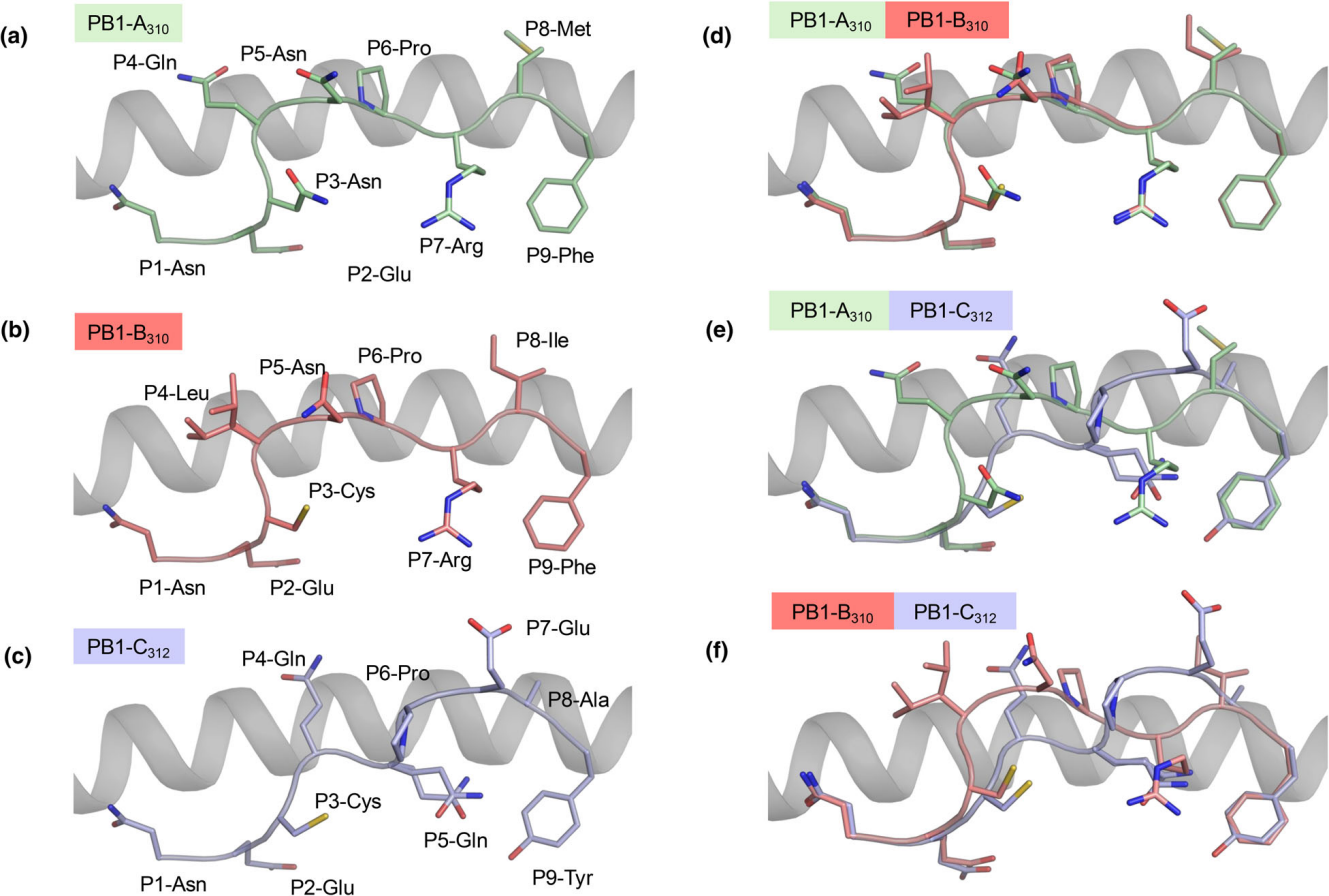
Crystal structures of the HLA‐B*18:01 binding cleft, presenting three PB1 derived influenza peptides. Crystal structure side view of the HLA‐B*18:01 binding cleft (grey cartoon) presenting PB1‐A_310_ (green sticks) **(a)**, PB1‐B_310_ (salmon) **(b)** and PB1‐C_312_ (light blue) **(c)**, with the peptides represented as cartoon backbones and stick sidechains. **(d)** Structural overlay of the peptide backbone and anchor positions only, for PB1‐A_310_, PB1‐B_310_ and PB1‐C_312_. The overlay with all visible side chains for PB1‐A_310_ and PB1‐B_310_ **(e)**, then PB1‐A_310_ and PB1‐C_312_ **(f)**.

**Figure 2:**
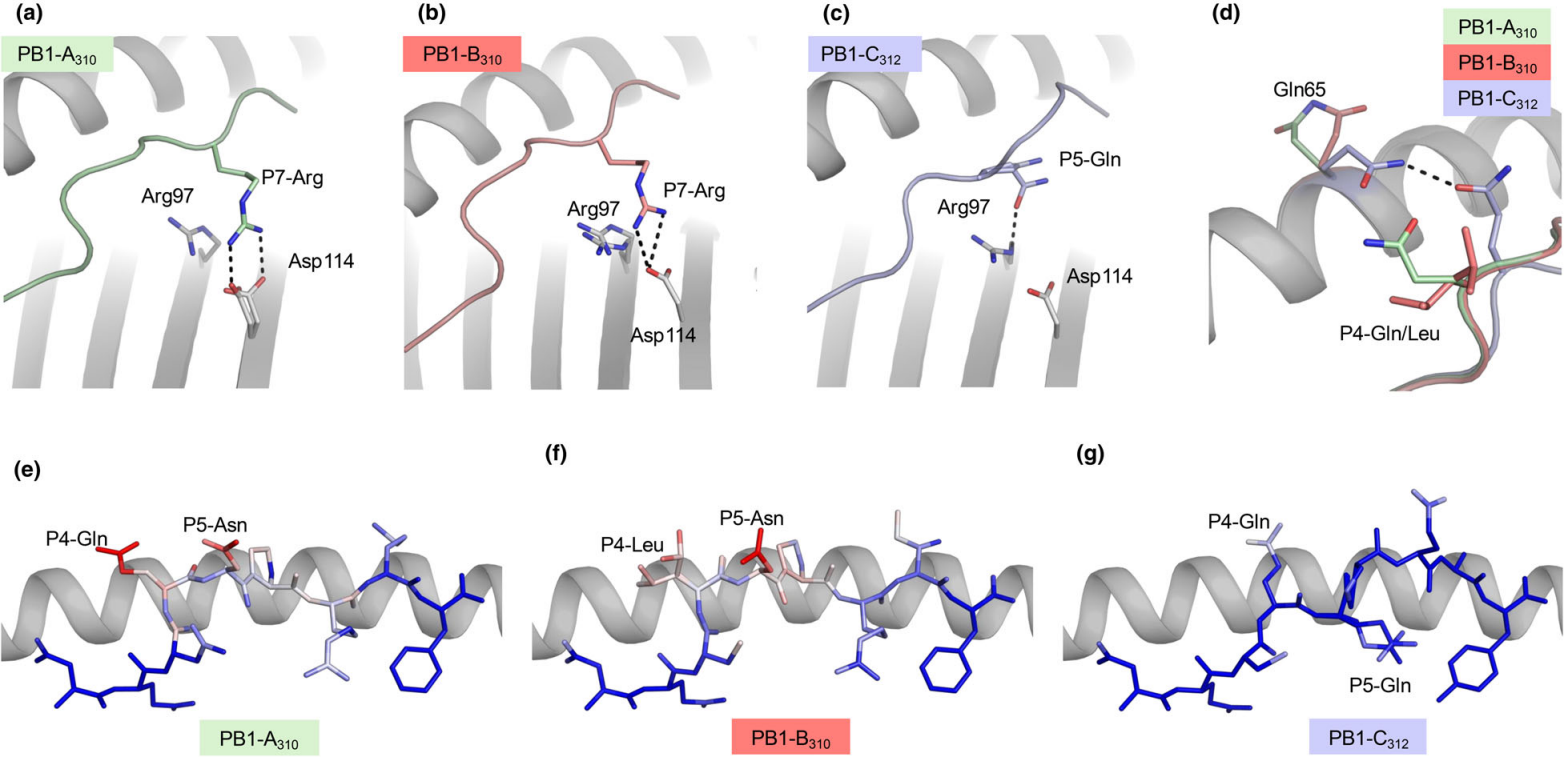
Structural characteristics of the PB1‐derived Influenza peptides presented by HLA‐B*18:01. Interactions between side chain residues Arg97 and Asp114 (sticks) of HLA‐B*18:01 (grey cartoon) presenting **(a)** PB1‐A_310_ (green sticks), **(b)** PB1‐B_310_ (salmon) and **(c)** PB1‐C_312_ (light blue), with hydrogen bonds shown as black dashes. **(d)** Overlay of the interaction between side Gln65 of HLA‐B*18:01 and the P4‐Gln/Leu of the PB1 derived peptides. B‐factor of PB1‐A_310_ **(e)** PB1‐B_310_ **(f)** and PB1‐C_312_ **(g)** represented as a blue, white and red colour spectrum (20 Å^2^ to 50 Å^2^).

In contrast, PB1‐C_312_ displays a different backbone conformation (Figure 1c, Supplementary figure 3c), yielding a r.m.s.d. value > 2.7 Å when aligned with either the PB1‐A_310_ (Figure 1e) or PB1‐B_310_ (Figure 1f). Conversely, PB1‐C_312_ has the P4‐Gln, P6‐Pro, P7‐Glu and P8‐Ala side chains solvent exposed (Figure 1c). The P5‐Gln faces into the D pocket acting as a secondary anchor, forming a hydrogen bond with Arg97 (Figure 2c). Unlike the P7‐Arg side chain for PB1‐A_310_ and PB1‐B_310_, P7‐Glu of PB1‐C_312_ would not be favoured as a secondary anchor because of the presence of Asp114 in the D pocket of HLA‐B*18:01.

The PB1‐C_312_ peptide was further stabilised by hydrogen bonds between P4‐Gln and Gln65 (Figure 2d), and P5‐Gln and Asn70 (Supplementary table 2). These peptide–HLA interactions were not present in the crystal structures with the PB1‐A_310_ and PB1‐B_310_ peptides (Supplementary table 2, Figure 2d). The peptide binding motif specific for HLA‐B*18:01 molecule according to NetMHCpan4.1^44^ does not show the presence of a secondary anchor residue in the middle of the peptides (Supplementary figure 4). However, the three peptides examined here, together with previously published HLA‐B*18:01‐restricted epitopes,^37^, ^41^, ^42^ collectively display a secondary anchor residue in the middle of the bound peptide either at P5 or P7 position (Table 1). Interestingly, the biochemical nature of the secondary anchor residues was diverse, ranging from a small cysteine to a large basic arginine residue (Table 1). The three most stable peptides, described so far, all share P5‐Ile as a secondary anchor residue (Table 1).

In addition, we observed multiple side‐chain conformations at P4‐Gln/Leu and P5‐Asn residues of PB1‐A_310_ and PB1‐B_310_ (Figure 1a and b, Supplementary figure 2) associated with higher B factors (Figure 2e and f) showing high movement of these residues. In contrast, PB1‐C_312_ adopted a rigid structure (Figure 2g) with a lower B factor value at P4‐Gln and the buried P5‐Gln (Figure 2g, Supplementary figure 2c–f). This might reflect the increased thermal stability observed for HLA‐B*18:01‐PB1‐C_312_ complex (Table 1). Notably, the peptide backbones conformations of PB1‐A_310_ and PB1‐B_310_ were different from previously solved peptide‐HLA‐B*18:01 crystal structures (Supplementary figure 3d, Table 1). Meanwhile, the backbone conformation of PB1‐C_312_ closely resembled that of the previously reported PB1_177‐B_, with a r.m.s.d. of 0.261 Å (Supplementary figure 3e). This structural similarity was surprising as the two peptides share only the P2‐Glu residue and PB1_177‐B_ has the lowest *T*_m_ observed for peptide‐HLA‐B*18:01 complexes, in contrast with PB1‐C_312_, which exhibits one of the highest *T*_m_ values (Table 1).

Overall, the data show that the PB1‐A_310_ and PB1‐B_310_ peptides adopted a similar conformation and have lower *T*_m_ values compared to the other published HLA‐B*18:01‐specific peptides.

### PB1‐A_310_ induces the highest IFNγ ^+^ CD8 ^+^ T cell response

Despite the differences in peptide presentation and stability, all peptides were able to be presented by the HLA‐B*18:01 molecule and have the potential to be recognised by CD8^+^ T cells. To investigate this, we next determined whether PB1‐A_310_, PB1‐B_310_ and PB1‐C_312_ peptides could induce a CD8^+^ T cell response by completing an intracellular cytokine staining (ICS) assay (Figure 3, Supplementary figures 5–7). HLA‐B*18:01^+^ PBMCs derived T cell lines (*n* = 5) showed that CD8^+^ T cells stimulated with the FLUAV‐derived PB1‐A_310_ peptide induced the highest level of IFNγ production with a mean of 1.19% ± 2.5% SD (Figure 3). A similar trend was observed for TNF, CD107a and MIP1β production (Supplementary figure 6). Interestingly, it is important to highlight that 4/5 HLA‐B*18:01^+^ samples responded weakly towards PB1‐A_310_ (average of 0.08% of IFNγ^+^ CD8^+^ T cells between 4 donors), while vacSG23 displayed a higher functional response (5.67% of IFNγ^+^ CD8^+^ T cells), which skewed the mean and standard deviation. This was subsequently followed by weaker response towards PB1‐B_310_ (mean IFNγ^+^ CD8^+^ T cells = 0.051% ± 0.079% SD) and PB1‐C_312_ (mean IFNγ^+^ CD8^+^ T cells = 0.0060% ± 0.013% SD).

**Figure 3:**
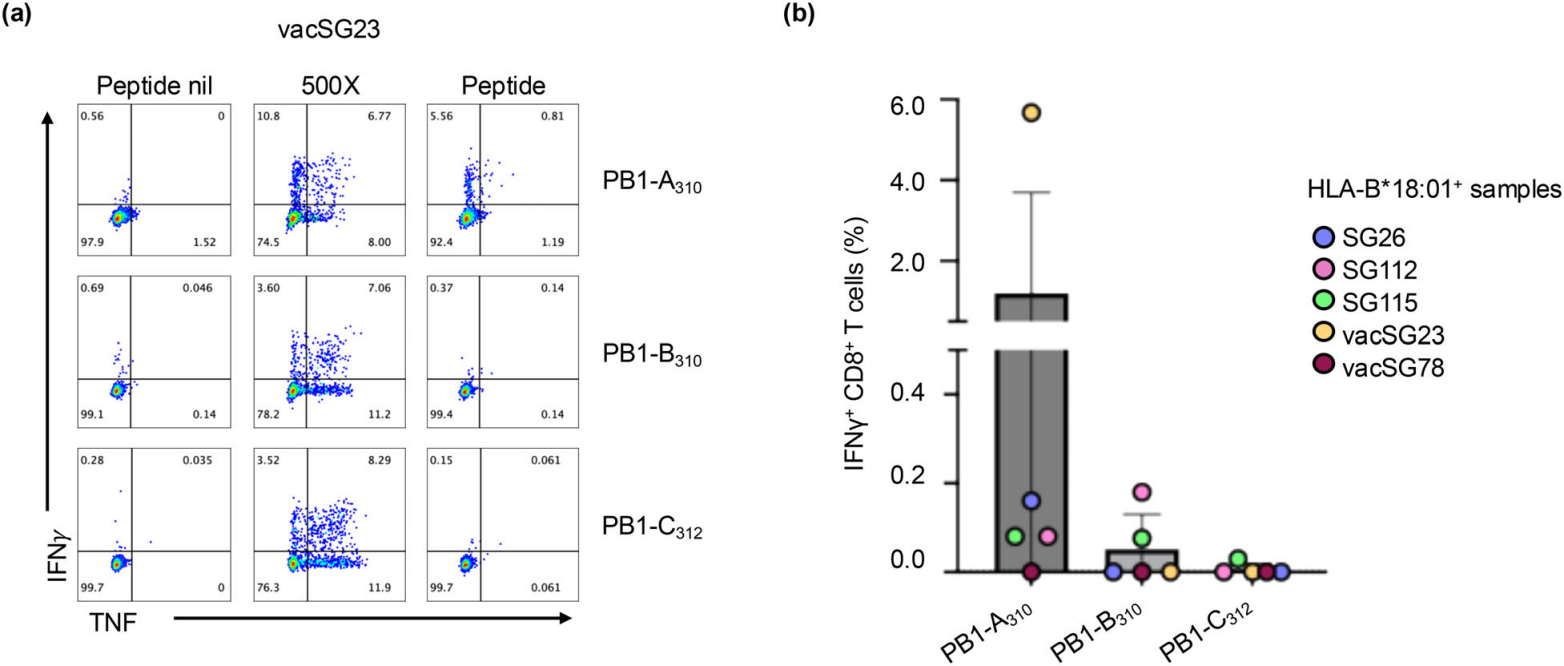
Intracellular cytokine staining of PB1‐A_310_, PB1‐B_310_ and PB1‐C_312_ specific T cell lines determining immunogenicity. PBMCs of HLA‐B*18:01^+^ cells were stimulated with either PB1‐A_310_, PB1‐B_310_ or PB1‐C_312_ at 10 μm for 10 days^+^ for T cell expansion (*n* = 5). After 10 days, an intracellular cytokine staining (ICS) assay was completed by restimulating each T cell line with the cognate peptide at 10 μm. A positive control 500× condition and nil peptide negative control was implemented for each T‐cell line. **(a)** Representative FACS plots of IFNγ vs TNF for vacSG23 are included. **(b)** A bar graph displaying mean ± SD of IFNγ^+^ CD8^+^ T cells % detected for each peptide via flow cytometry and gated as per Supplementary figure 5. Each coloured dot represents a donor's T‐cell response, while the bars illustrate the mean for each peptide.

Though moderate to weak CD8^+^ T cell responses were observed for PB1‐A_310_, PB1‐B_310_, and PB1‐C_312_, these results show that the three homologous peptides are immunogenic at varying levels.

### Minimal T cell cross‐reactivity identified to PB1‐derived peptides

Despite the variable moderate to weak immunogenicity of the homologous peptides, we next assessed their capacity to induce cross‐reactive CD8^+^ T cell responses. To evaluate this, CD8^+^ T cell lines pulsed with each peptide were generated (*n* = 5) and were then restimulated with each of the three homologous peptides (Figure 4 and Supplementary figure 7).

**Figure 4:**
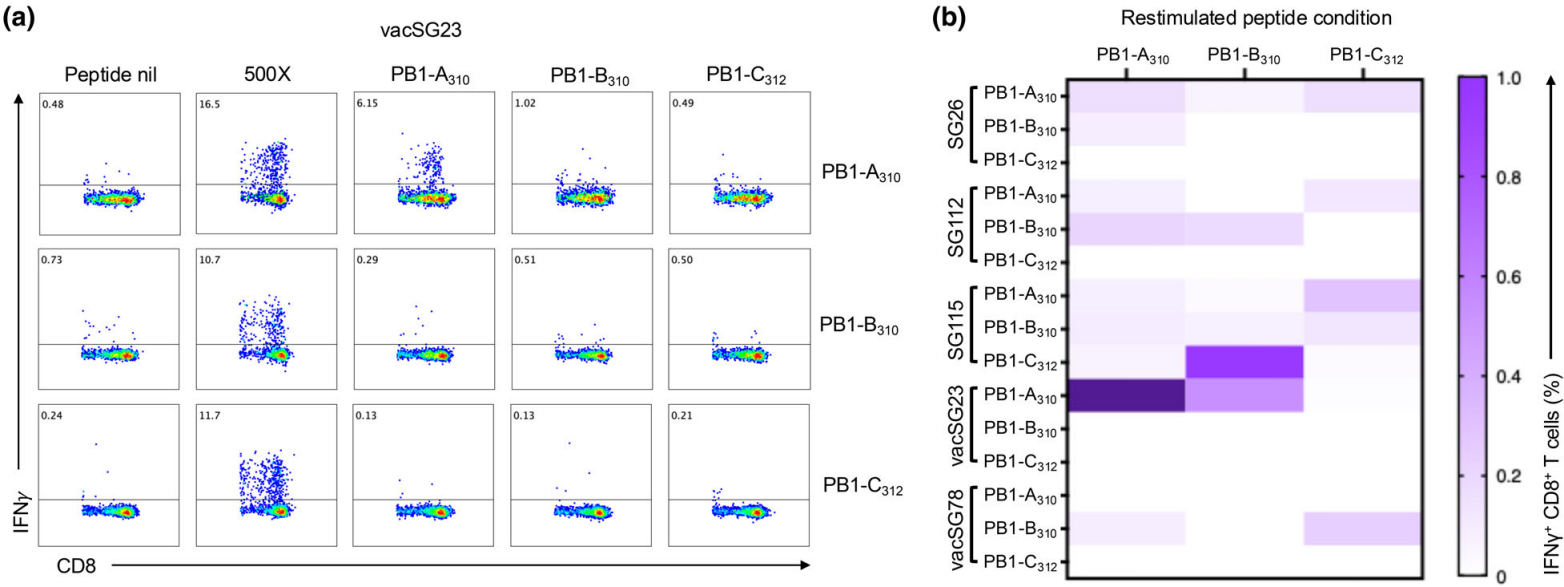
Cross‐reactivity heatmap of PB1‐A_310_, PB1‐B_310_ and PB1‐C_312_ peptides. PB1‐A_310_, PB1‐B_310_ and PB1‐C_312_ T cell lines were generated from HLA‐B*18:01^+^ typed PBMCs of previously used samples (*n* = 5). An intracellular cytokine staining assay was completed to assess T‐cell reactivity and cross‐reactivity for each peptide‐stimulated line and condition. **(a)** vacSG23 was chosen as the representative for IFNγ^+^/CD8^+^ T cells FACS plots. **(b)** A heat map outlining the production of IFNγ^+^ by CD8^+^ T cells, above the nil peptide control, and the gradient colour represents the amount of IFNγ produced from T cells (from 0 to 1%) gated as per Supplementary figure 5. The darker cells outline the proportion of IFNγ^+^ CD8^+^ T cells >1%.

PB1‐A_310_–pulsed CD8^+^ T cells demonstrated the highest frequency of specificity with positive IFNγ^+^ CD8^+^ T cell responses towards the PB1‐A_310_ peptide seen in 4/5 donors. Similarly, PB1‐A_310_–pulsed CD8^+^ T cells demonstrated the highest frequency of cross‐reactive responses, with positive IFNγ^+^ CD8^+^ T cell responses seen towards 7/10 cross‐reactive conditions (Figure 4 and Supplementary figure 7). In vacSG23, the sample with the highest cognate response (5.67% IFNγ^+^ CD8^+^ T cells), strong T cell cross‐reactivity was seen towards PB1‐B_310_, (0.54% IFNγ^+^ CD8^+^ T cells). T‐cell cross‐reactivity was also seen towards the PB1‐C_312_ peptide but at a lower level (0.01% IFNγ^+^ CD8^+^ T cells). Although responses were minimal, a similar trend was seen in SG26 and SG115 samples, where PB1‐A_310_–pulsed T cells responded towards both PB1‐B_310_ (0.06 and 0.03% IFNγ^+^ CD8^+^ T cells, respectively) and PB1‐C_312_ (0.16 and 0.30% IFNγ^+^ CD8^+^ T cells, respectively), while SG112 samples displayed some T cell cross‐reactivity towards the PB1‐C_312_ peptide (0.12% IFNγ^+^ CD8^+^ T cells, respectively).

PB1‐B_310_ pulsed CD8^+^ T cell lines had less frequent specificity than their PB1‐A_310_ peptide pulsed counterparts, with IFNγ^+^ CD8^+^ T cell responses towards the cognate PB1‐B_310_ peptide in lines derived from 2/5 donors (Figure 4 and Supplementary figure 7). Interestingly, CD8^+^ T cell cross‐reactivity was observed in 6/10 cross‐reactive conditions. This included limited responses towards both PB1‐A_310_ and PB1‐C_312_ in SG115 sample (0.10% and 0.13% IFNγ^+^ CD8^+^ T cells, respectively) and vacSG78 sample (0.09% & 0.24% IFNγ^+^ CD8^+^ T cells, respectively), or PB1‐A_310_ only, in samples from SG26 and SG112 (0.09% and 0.21% IFNγ^+^ CD8^+^ T cells, respectively).

Conversely, PB1‐C_312_‐pulsed CD8^+^ T cell lines displayed minimal specificity to the cognate peptide in cells from only 1/5 donors, namely SG115 (Figure 4 and Supplementary figure 7). CD8^+^ T cells from this donor sample displayed some T cell cross‐reactivity, with IFNγ^+^ CD8^+^ T cell responses observed towards both PB1‐A_310_ (0.06% IFNγ^+^ CD8^+^ T cells) and PB1‐B_310_ (0.96% IFNγ^+^ CD8^+^ T cells) that are notably higher than the response to the cognate peptide.

Notably, although the PB1‐C_312_ peptide activated minimal CD8^+^ T cell responses, cross‐reactivity was observed towards the PB1‐C_312_ in 6/10 cross‐reactive conditions across samples from all (5/5) donors, suggestive of previously activated CD8^+^ T cells being able to exhibit single‐directional cross‐reactivity. Comparatively, there was some cross‐reactivity towards PB1‐A_310_ in 5/10 conditions across samples from 4/5 donors, and PB1‐B_310_ in 4/10 conditions in samples from 3/5 donors.

Overall, cognate‐specific and cross‐reactive CD8^+^ T cell responses towards PB1‐A_310_, PB1‐B_310_, and PB1‐C_312_ were varied and mostly weak, with some evidence of single‐directional cross‐reactivity towards PB1‐C_312_.

## Discussion

CD8^+^ T cells play a critical role in the clearance of influenza infections^45^ and contribute to long‐lived immunity through memory T cells,^21^, ^46^ which can persist for decades and be reactivated by subsequent infections.^47^ This makes CD8^+^ T cells an attractive target for next‐generation vaccine design, with several candidates currently undergoing clinical evaluation, targeting either internally conserved or HA proteins.^48^, ^49^, ^50^, ^51^ However, the outcomes of the clinical trials have been so far variable, highlighting the need for a deeper understanding of the mechanisms underlying CD8^+^ T cell cross‐reactivity. Previous studies have demonstrated that cross‐reactive CD8^+^ T cells can recognise homologous influenza‐derived peptides across different HLA allotypes.^34^, ^38^, ^43^, ^52^, ^53^ Therefore, this study investigated this phenomenon in the context of HLA‐B*18:01, an allele expressed in over 180 million people, spanning multiple continents around the world.^36^

There are several published structures of HLA‐B*18:01 in complex with a variety of peptides^37^, ^38^, ^41^, ^42^, ^43^ that display a range of immunogenicity's, from the highly immunogenic NP_219_ peptide^37^ to the weakly immunogenic PB1_177‐B_ peptide.^38^ Interestingly, these epitopes all adopt a similar peptide backbone in the cleft of HLA‐B*18:01. In this study, we found that the PB1‐C_312_ peptide also displayed a similar conformation to the published peptide‐HLA‐B*18:01 structures, and closely resembled to the non‐immunogenic PB1_177‐B_ peptide in complex with HLA‐B*18:01.^38^ Similarly, we found that the PB1‐C_312_ peptide was at most, weakly immunogenic in the samples from HLA‐B*18:01^+^ samples.

Conversely, PB1‐A_310_ and PB1‐B_310_ adopt a similar conformation to each other, but distinct from PB1‐C_312_, and other published peptide‐HLA‐B*18:01 structures^37^, ^38^, ^41^, ^42^ representing a novel peptide conformation within this allomorph. Interestingly, PB1‐A_310_ and PB1‐B_310_ were the most immunogenic in our study, with the PB1‐A_310_ peptide displaying the highest immunogenicity, followed by the PB1‐B_310_ peptide. This suggests increased peptide backbone conformational diversity within the HLA‐B*18:01 binding cleft. Additional examples will be required to determine whether this feature is shared by other immunogenic peptides restricted to HLA‐B*18:01.

When comparing the structures of the three peptides, the PB1‐C_312_ contains a different PΩ primary anchor residue (P9‐Tyr) compared to PB1‐A_310_ and PB1‐B_310_ (P9‐Phe). Interestingly, all three peptides harbour a central secondary anchor residue (P7‐Arg in PB1‐A_310_ and PB1‐B_310_ and a P5‐Gln in PB1‐C_312_), not described in the HLA‐B*18:01 peptide binding motif^44^ likely because of the lack of residue conservation (C, R, I, F, T, Q) and position (5 or 7). The observation that the three most stable peptides all shared a P5‐Ile secondary anchor is interesting, and more data will be required to confirm if this is a feature of HLA‐B*18:01 molecule. Further, the PB1‐C_312_ displayed the highest pHLA stability, despite being associated with the weakest CD8^+^ T cell response. This shows that higher peptide‐HLA stability does not translate to higher T cell activation and highlights the current challenges in predicting epitope specific responses.

CD8^+^ T cell responses to peptides can be variable between donors and differ based on factors such as previous infection history, immunisation, T cell repertoire and HLA profile.^54^, ^55^, ^56^, ^57^, ^58^, ^59^ Therefore, it was unsurprising to see that the CD8^+^ T cell responses towards the three homologous peptides were variable between samples derived from different donors. Further, multiple studies have demonstrated that cross‐reactive influenza specific TCRs preferentially engage conserved structural features of the pHLA complex, including peptide backbone conformation and conserved side chains.^29^, ^43^, ^60^ In line with these findings, we saw CD8^+^ T cell cross‐reactivity towards the structurally similar PB1‐A_310_ and PB1‐B_310_ peptides. It is likely that this T cell cross‐reactivity is mediated by TCR recognition of the conserved solvent exposed regions of P5‐Asn and P6‐Pro in combination with the similar backbone conformation of the two peptides. Interestingly, CD8^+^ T cell lines stimulated with the PB1‐A_310_ (*n* = 4/5 donors) or PB1‐B_310_ peptides (*n* = 2/5 donors) demonstrated minimal cross‐reactivity towards the weakly immunogenic PB1‐C_312_ peptide, despite CD8^+^ T‐cell lines pulsed with the PB1‐C_312_ peptide displaying minimal CD8^+^ T cell responses. This single‐direction T cell cross‐reactivity suggests that the CD8^+^ T cells already activated by the immunogenic PB1‐A_310_ and PB1‐B_310_ peptides have less specificity and potentially also suggests a lower threshold for activation.

In this study, we have expanded the current knowledge on HLA‐B*18:01 peptide presentation, solving the first crystal structure of HLA‐B*18:01 presenting a FLUCV‐derived peptide and evaluated CD8^+^ T‐cell responses and cross‐reactivity towards three homologous PB1‐derived peptides. Although T cell cross‐reactivity was variable, the structural similarity between the PB1‐A_310_ and PB1‐B_310_ combined with a higher immunogenicity of PB1‐A_310_ epitope appears to lead to a more frequent T cell cross‐reactivity. This suggests that structural similarity of peptides in this case underpinned T cell cross‐reactivity. In addition, we identify the presence of secondary anchor residue within the HLA‐B*18:01‐restricted peptide, with a shared P5‐Ile in the most stable peptides.

However, the limited CD8^+^ T cell response and cross‐reactivity towards the distinct PB1‐derived epitopes are unlikely to produce broad protection against the different influenza types, within HLA‐B*18:01^+^ individuals. Overall, this study provides new insights into the structural and immunological features of T cell epitopes especially across influenza viruses.

## Methods

### Protein expression and purification

The HLA transmembrane domain region was removed, and the cDNA coding sequence was sub‐cloned into the pET30 plasmid vector (GenScript, Piscataway, USA). BL21‐RIL *Escherichia coli* cells were used to express the recombinant HLA heavy‐chain protein and the human β2‐microglobulin (β2m) as inclusion bodies.^61^ HLA‐B*18:01 was then refolded with 5 mg of either the PB1‐A_310_, PB1‐B_310_ or PB1‐C_312_ peptides with 30 mg of HLA heavy chain and 10 mg of β2m in a refold buffer with 3 m urea (ThermoFisher, Melbourne, Australia), 0.4 m L‐arginine (Merck, Darmstadt, Germany), 0.1 m Tris–HCl pH 8.0 (ThermoFisher, Melbourne, Australia), 2 mm Na‐EDTA pH 8.0 (VWR chemicals, Leuven, Belgium), 0.16% w/v reduced glutathione (Goldbio, St Louis, USA) and 0.03% w/v oxidised glutathione (Goldbio, St Louis, USA). The refolding solution was dialysed against 10 mm Tris–HCl pH 8.0 (ThermoFisher, Melbourne, Australia), and the peptide‐HLA‐B*18:01 complexes were purified via anion exchange chromatography utilising diethylaminomethyl cellulose (DEAE) resin followed by HiTrap Q (Cytiva, Marlborough, USA).

### Protein crystallisation, structure determination and refinement

Purified peptide‐HLA‐B*18:01 complexes at 6 mg.mL^−1^ were crystallised via vapour diffusion, using a sitting drop method at 20°C.^61^ The crystal conditions were for HLA‐B*18:01‐PB1‐A_310_: 0.2 m Ammonium Acetate, 0.1 m Bis‐Tris pH 7.5% and 25% PEG3350 (Hampton Research, Aliso Viejo, USA); for HLA‐B*18:01‐PB1‐B_310_: 0.05 m Lithium acetate (Hampton Research, Aliso Viejo, USA), 20% PEG3350 (Sigma‐Aldrich, St. Louis, USA), 10 mm MgCl_2_ (Sigma‐Aldrich, St. Louis, USA); and for HLA‐B*18:01‐PB1‐C_312_: 0.1 m Bis‐Tris pH 6.5 and 0.5 m Magnesium formate dihydrate (Hampton Research, Aliso Viejo, USA). Crystals were cryoprotected in a 1:3 ratio of mother liquor:20% Ethylene glycol (Hampton Research, Aliso Viejo, USA) and flash‐frozen in liquid nitrogen. Data collection was done at the Australian Synchrotron MX2 beamline and processed using XDS.^62^ The structures were solved by molecular replacement using PHASER v.2.8.3^63^ in CCP4 suite v8.0.005 software^64^ with HLA‐B*18:01 model (PDB: 6MT3^43^) without the peptide as the search template. Models were built in the COOT v0.9.8.4 software^65^ and refined using PHENIX v1.20‐1‐4487‐000.^66^ Validation and deposition of the final structures were then carried out on the PDB OnDep System, under the accession numbers: 9NN7 for HLA‐B*18:01 PB1‐A_310_, 9ZZ8 for HLA‐B*18:01 PB1‐B_310_ and 9ZZN for HLA‐B*18:01 PB1‐C_312_. All figures were generated using PyMoL (v2.5).

### Differential scanning fluorimetry (DSF)

Thermal stability measurements for HLA‐B*18:01 presenting PB1‐A_310_, PB1‐B_310_ and PB1‐C_312_ peptides were undertaken using The ViiA 7 real‐time PCR system (ThermoFisher, Melbourne, Australia) using the TAMRA reporter (x3 − m3 filter). The HLA‐A*02:01 refolded with an influenza‐derived peptide (^50^GILGFVFTL^58^) was used as a positive control.^26^ Each protein sample were used at 5 and 10 μm with duplicates for each concentration with 10× SYPRO Orange (ThermoFisher, Melbourne, Australia). Each sample was then heated from 25°C to 95°C at a rate of 1°C per min. The excitation wavelength set to ~550 nm with an emission detection reading at ~587 nm was measured at every increase of 0.5°C. Data were normalised using GraphPad Prism 9 (v9.3) and the temperature where fluorescence intensity reached 50% of its maximum amount was determined as the melting temperature or *T*_m_.

### Peripheral blood mononuclear cell (PBMC) isolation

PBMCs from HLA‐B*18:01^+^ individuals were harvested and used to generate PB1‐A_310_, PB1‐B_310_ and PB1‐C_312_ pulsed T cell lines. PBMCs were harvested from Buffy coats acquired from the Australian Red Cross Life Blood and from whole peripheral blood donations from the laboratory's biobank following informed consent from the donors. PBMCs were isolated using the Ficoll density gradient centrifugation method as previously descried.^43^, ^67^ The HLA genotyping was performed by AlloSeq Tx17 (CareDx Pty) using All Type NGS high‐resolution genotyping on the IonTorrent NGS platform or by the Department of Clinical Immunology and PathWest at Fiona Stanley Hospital, Murdoch, Australia. Donor information can be found in Supplementary table 3. All research was approved by the La Trobe University Human Ethics Committee (Approval No: HEC21097) and undertaken in accordance with the declaration of Helsinki.

### Generation of peptide pulsed CD8^+^ T cell lines

Peptides with > 80% purity were synthesised from GenScript Biotech (Piscataway, USA). CD8^+^ T‐cell lines were generated using our published method,^68^ with either PB1‐A_310_, PB1‐B_310_ or PB1‐C_312_ peptide and using HLA‐B*18:01^+^ PBMCs. Only one‐third of PBMCs were peptide pulsed with 10 μM of PB1‐A_310_, PB1‐B_310‐318_ or PB1‐C_312_ peptide and were incubated for 1.5 h at 37°C with 5% CO_2_. After 1.5 h, peptide‐treated cells were washed twice and added to the remaining two‐thirds of unstimulated PBMCs for expansion. The PB1‐A_310_, PB1‐B_310_ and PB1‐C_312_‐pulsed T cell lines were then cultured for ~10 days in RPMI‐1640 (Gibco, New York, USA) supplemented with 1× non‐essential amino acids (100× NEAA) (Gibco, New York, USA), 1× of Penicillin Streptomycin Glutamine (Gibco, New York, USA), 5 mm of HEPES (Sigma‐Aldrich, St. Louis, USA), 2 mm of L‐glutamine (Sigma‐Aldrich, St. Louis, USA), foetal calf serum (FCS) (Sigma‐Aldrich, St. Louis, USA) and 50 μm of β‐mercaptoethanol (Sigma‐Aldrich). 10 IU mL^−1^ of recombinant human IL2 (Peprotech, Rocky Hill, USA) was supplemented on the fourth and seventh day or every 3 days after until required.

### Intracellular cytokine staining (ICS) of peptide pulsed CD8^+^ T cell lines

ICS was completed on PB1‐A_310_, PB1‐B_310_ and PB1‐C_312_ pulsed CD8^+^ T cell lines to observe whether CD8^+^ T cells could respond to their cognate peptide and cross‐react against each other peptide. First, PB1‐A_310_, PB1‐B_310_ and PB1‐C_312_ pulsed CD8^+^ T cell lines were counted using the trypan blue exclusion method to obtain 1.5 × 10^5^ cells/well. CD8^+^ T cell lines were stimulated with 10 μm of PB1‐A_310_, PB1‐B_310_ and PB1‐C_312_ peptide individually. A positive control Cell Stimulation Cocktail 500× (eBiosciences, San Diego, USA) and a nil peptide R0 only negative control were added for each cell line. All cell lines were then incubated for 5 h at 37°C with 5% CO_2_ with 1:200 of anti‐human CD107a‐AF488 (eBiosciences, San Diego, USA), 1:1400 Golgi‐stop (BD Biosciences, Franklin Lakes, USA) and 1:1000 Golgi‐Plug (BD Biosciences, Franklin Lakes, USA) for each cell condition. Each condition was surface stained after the 5 h incubation for 30 min at 4°C with anti‐human CD3‐BV480 (1:100; BD Biosciences, Franklin Lakes, USA), anti‐human CD4‐BV650 (1:100; BD Biosciences, Franklin Lakes, USA), anti‐human CD8‐PerCPCy5.5 (1:50; BD Biosciences, Franklin Lakes, USA), anti‐human CD19‐APCH7 (1:100; BD Biosciences, Franklin Lakes, USA), anti‐human CD14‐APCH7 (1:100; BD Biosciences, Franklin Lakes, USA) and Live/Dead‐NIR (1:1000; Molecular Probes, Eugene, USA). All cells were subsequently washed with PBS and then fixed and permeabilised with BD‐Fix Perm buffer (BD Biosciences, Franklin Lakes, USA) for 20–30 min at 4°C. Cells were then washed with Perm‐wash buffer (BD Biosciences, Franklin Lakes, USA) and intracellularly stained with anti‐human IFNγ‐BV421 (1:100; BD Biosciences, Franklin Lakes, USA), anti‐human TNF‐PeCy7 (1:100; BD Biosciences, Franklin Lakes, USA), anti‐human MIP1β‐APC (1:100; BD Biosciences, Franklin Lakes, USA) diluted in Perm‐wash buffer (BD Biosciences, Franklin Lakes, USA) for 30 min at 4°C in the dark. All samples were acquired using the BD FACSymphony A3 analyser (BD Biosciences, Franklin Lakes, USA) and analysed using FlowJo (BD Biosciences, Franklin Lakes, USA) version 10.10.0, as per Supplementary figure 5.

## Author contributions

**Lawton D Murdolo:** Investigation; formal analysis; writing – original draft; writing – review and editing; project administration; data curation; funding acquisition. **Samuel Liwei Leong:** Investigation; formal analysis; project administration; writing – review and editing; writing – original draft; data curation; funding acquisition. **Janesha C Maddumage:** Writing – original draft; writing – review and editing; supervision; validation; project administration. **Lloyd D'Orsogna:** Validation; project administration; investigation; methodology; writing – review and editing. **Demetra SM Chatzileontiadou:** Validation; project administration; supervision; writing – review and editing; writing – original draft. **Emma J Grant:** Conceptualization; formal analysis; supervision; project administration; funding acquisition; writing – original draft; writing – review and editing; data curation; validation. **Stephanie Gras:** Conceptualization; funding acquisition; writing – original draft; writing – review and editing; formal analysis; project administration; supervision; data curation; validation.

## Conflict of interest

The authors declare no conflict of interest.

## Ethics statement

All research was approved by the La Trobe University Human Ethics Committee (Approval No: HEC21097) and undertaken in accordance with the declaration of Helsinki.

## Consent statement

Human Peripheral Blood Mononuclear cells (PBMCs) were obtained from Buffy coats acquired from the Australian Red Cross Life Blood, where donors provided consent for unused materials at the time of donation. PBMCs were also obtained from whole peripheral blood donations, following written informed consent from volunteers.

## Supporting information


Supplementary figures 1–7

Supplementary tables 1–3

## Data Availability

The crystal structure data are openly available in the Protein Data Bank (https://www.rcsb.org/) and PDB code accession numbers 9ZZ7, 9ZZ8 and 9ZZN as indicated in Table 1. All other data that support the findings of this study are available from the corresponding author, SG, upon reasonable request.
