# Supplementary material for: Assessing CD8+ T cell cross‐reactivity to influenza A, B and C virus‐derived peptides presented by HLA‐B*18:01
Source: Clin Transl Immunology. 2026 Oct 13;15(10):e70123. doi: 10.1002/cti2.70123 (PMC13659276; doi:10.1002/cti2.70123)

Supplementary Figure 1

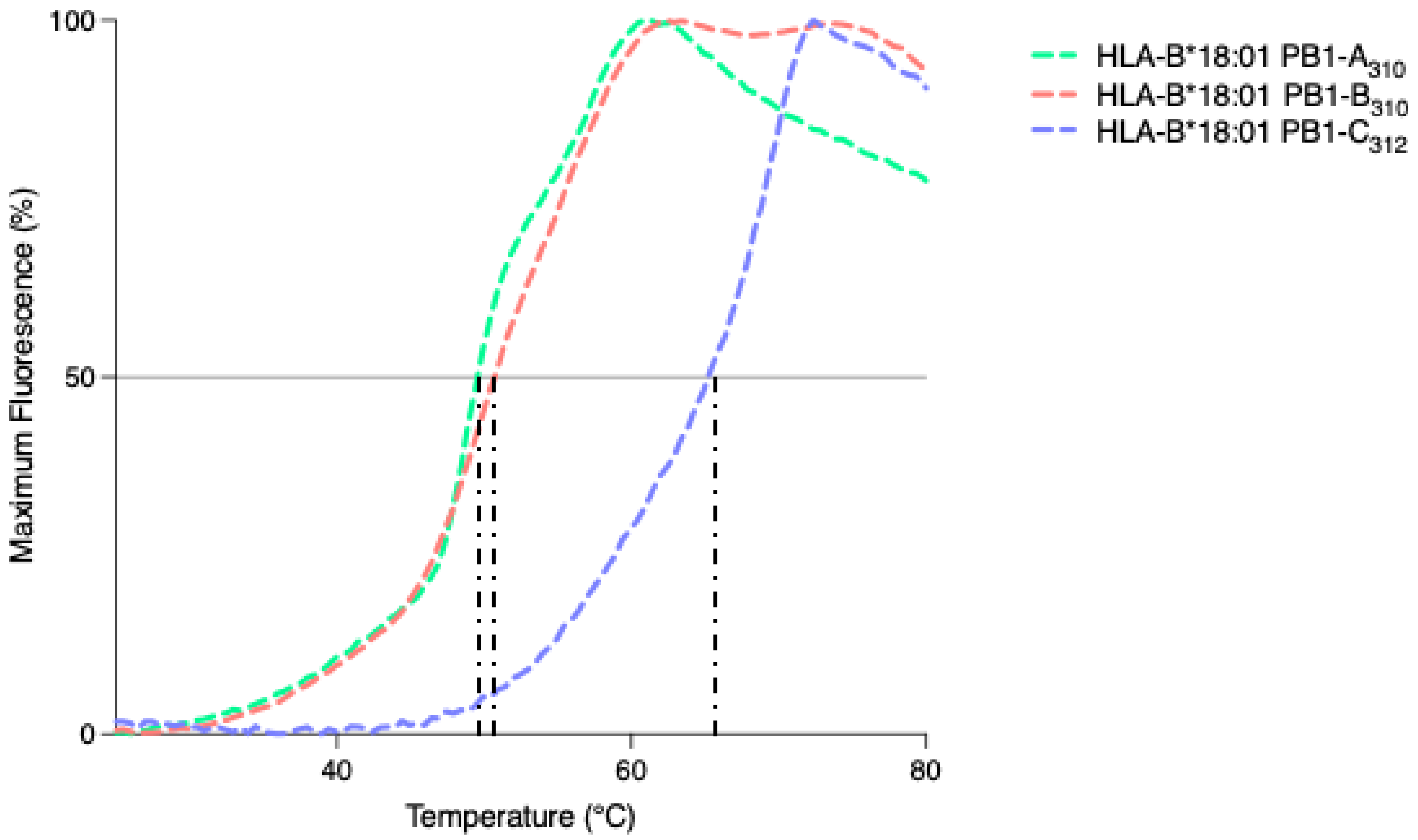

Supplementary Figure 2

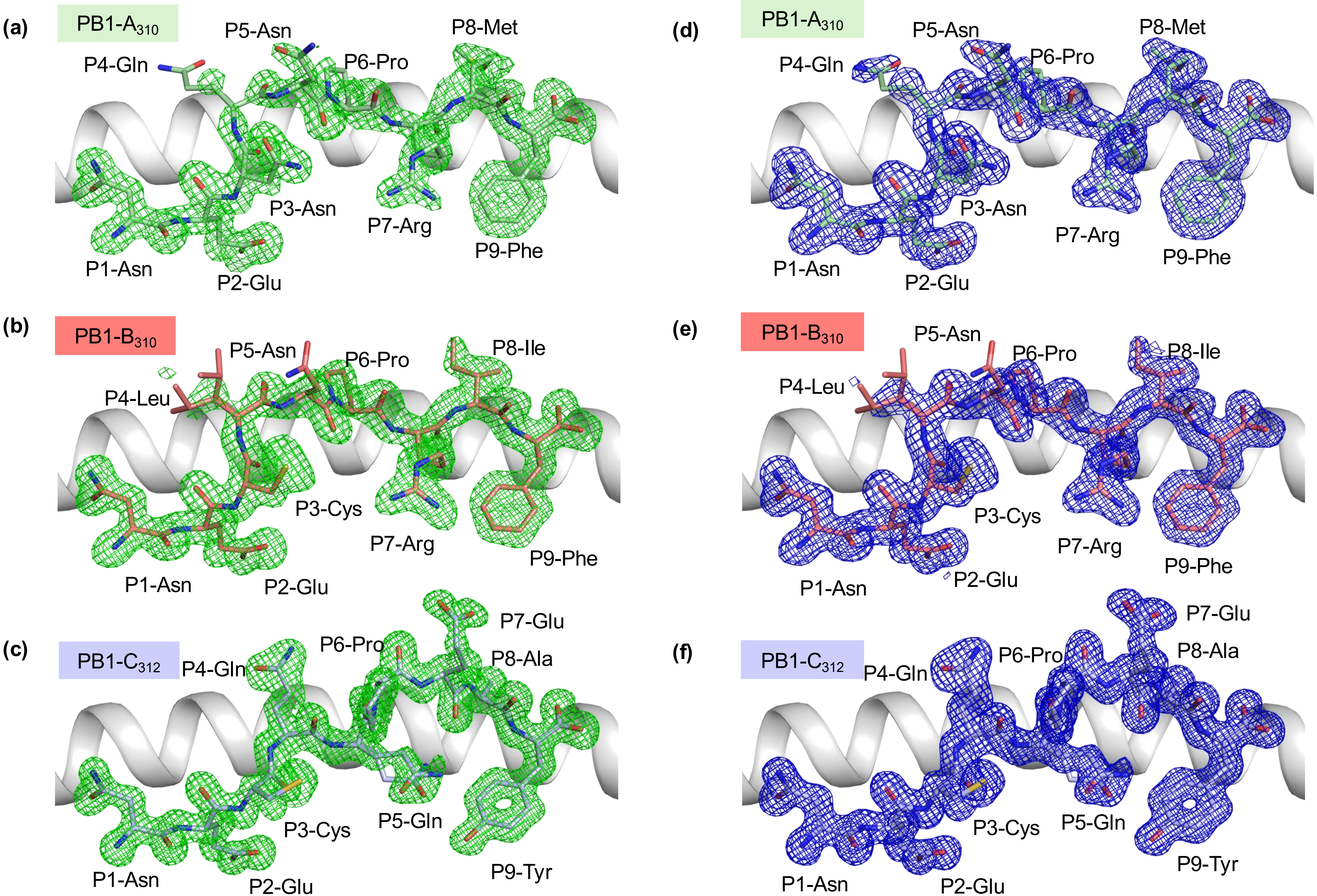

Supplementary Figure 3

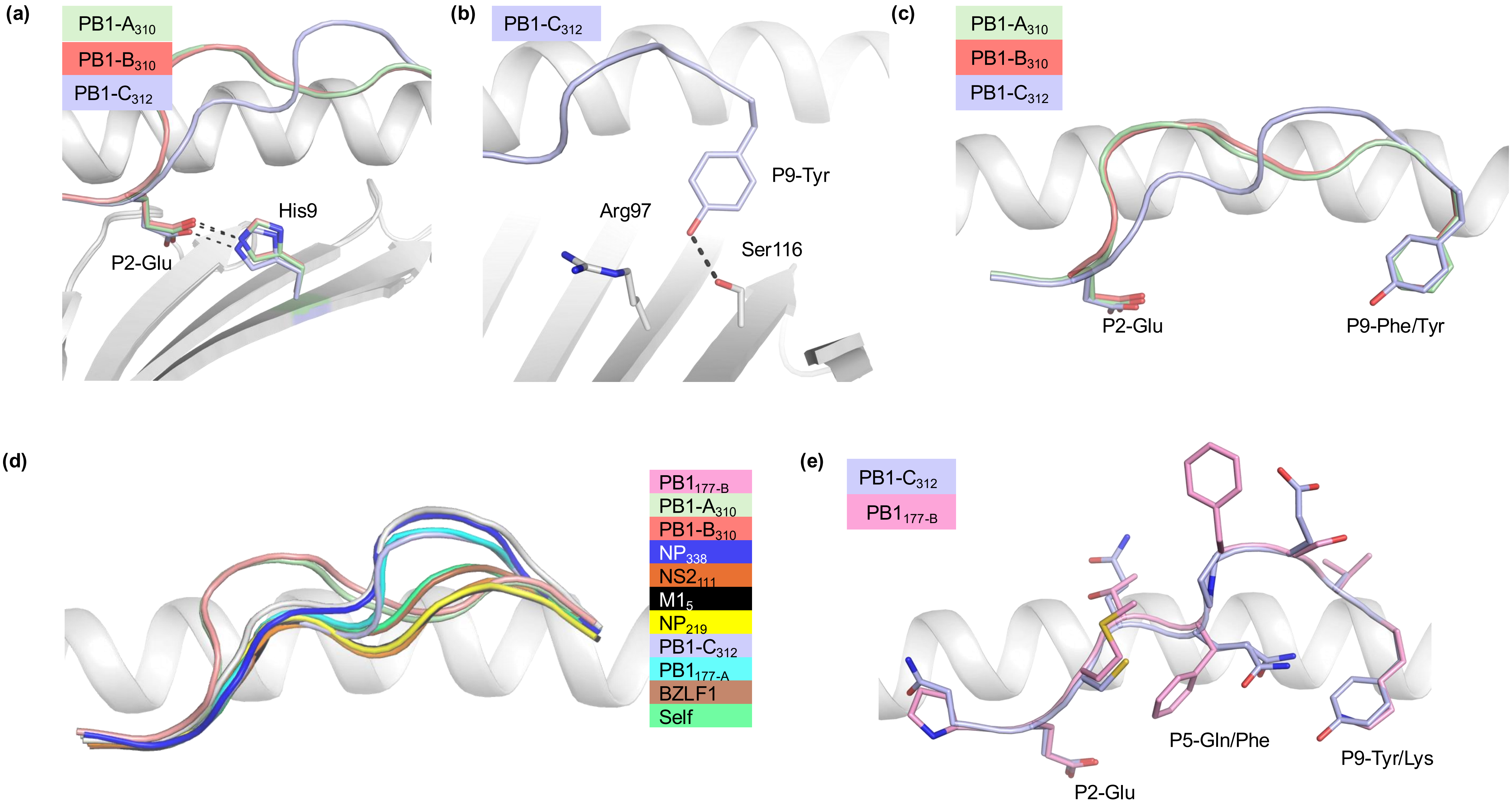

Supplementary Figure 4

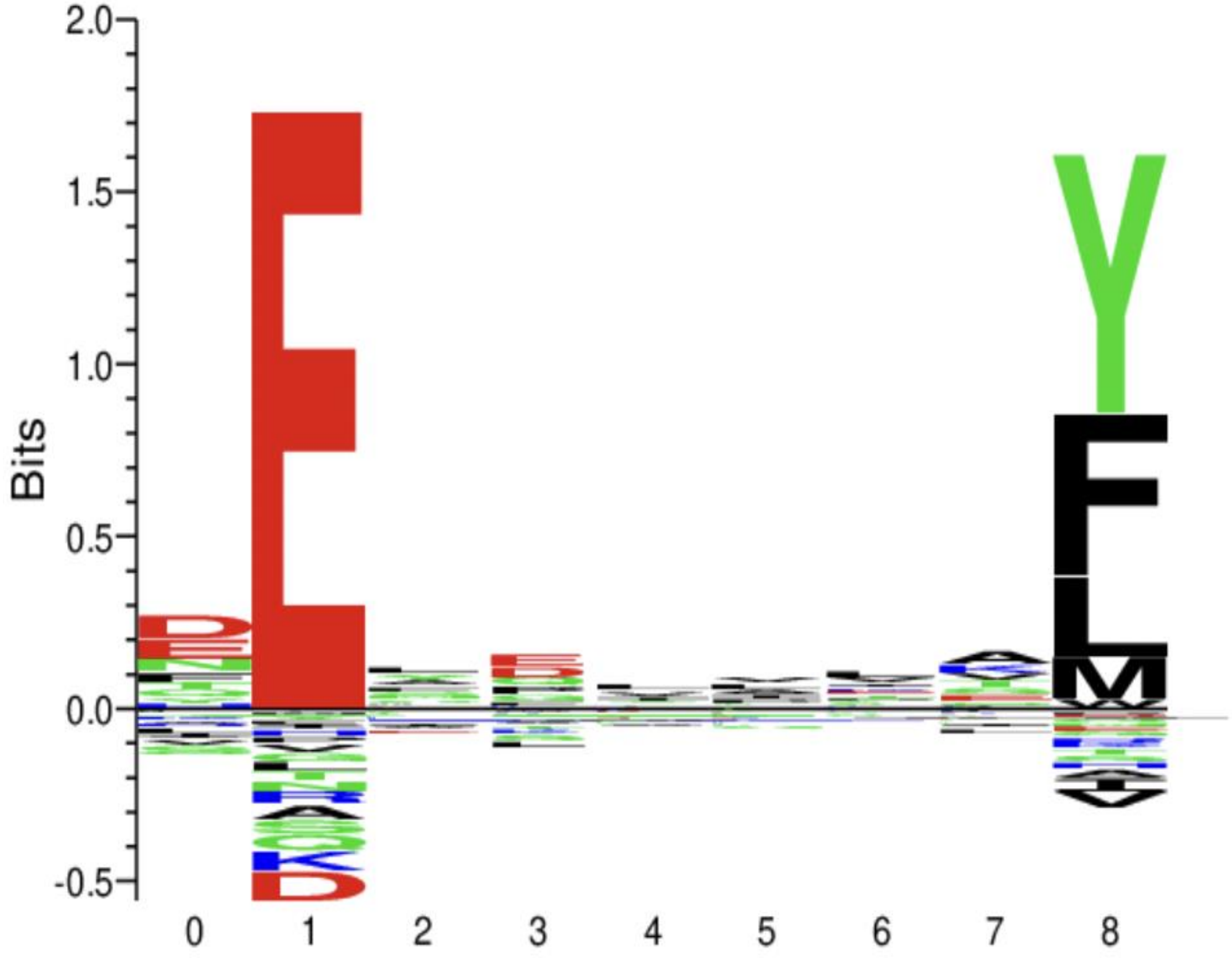

Supplementary Figure 5

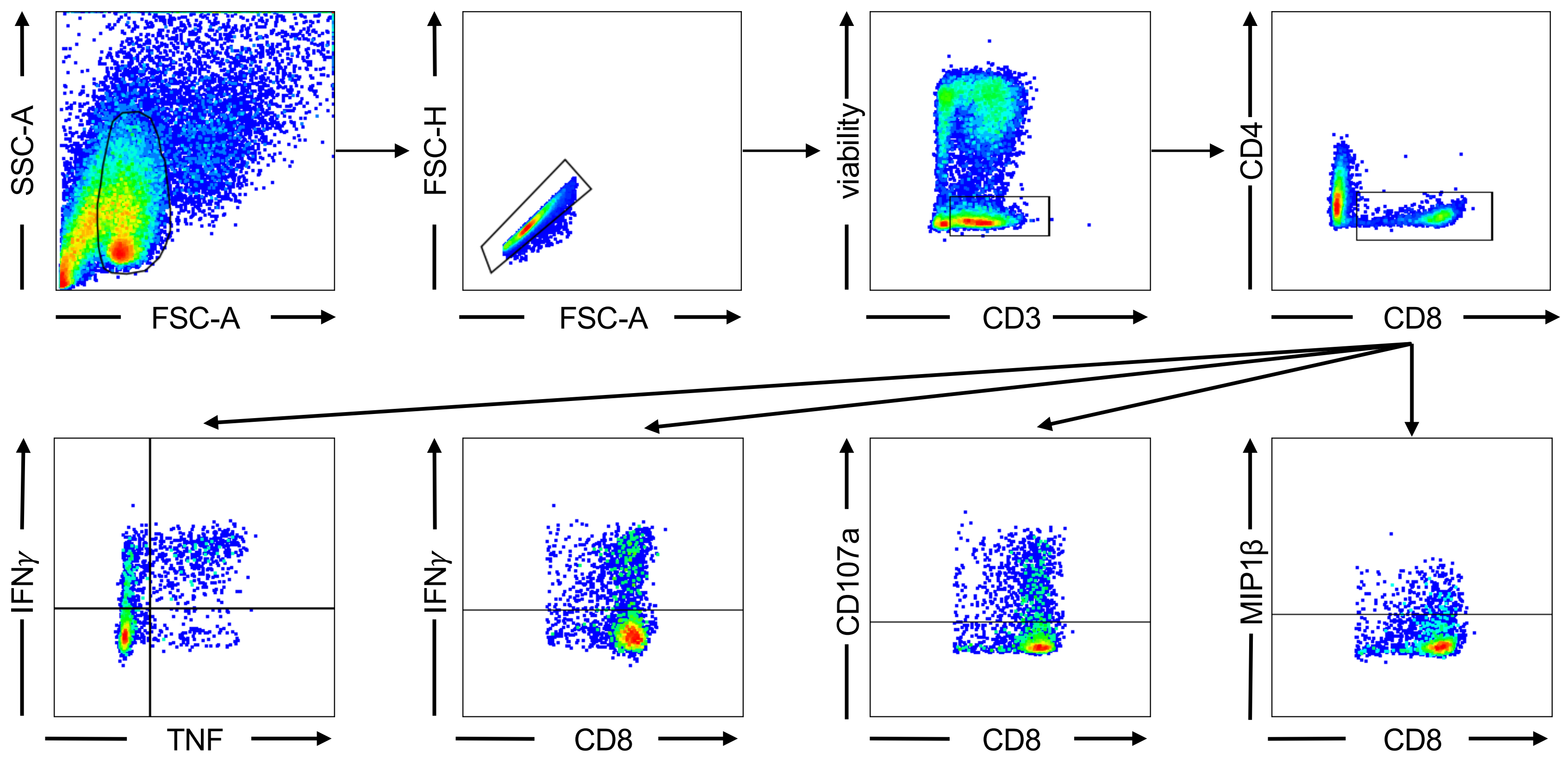

Supplementary Figure 6

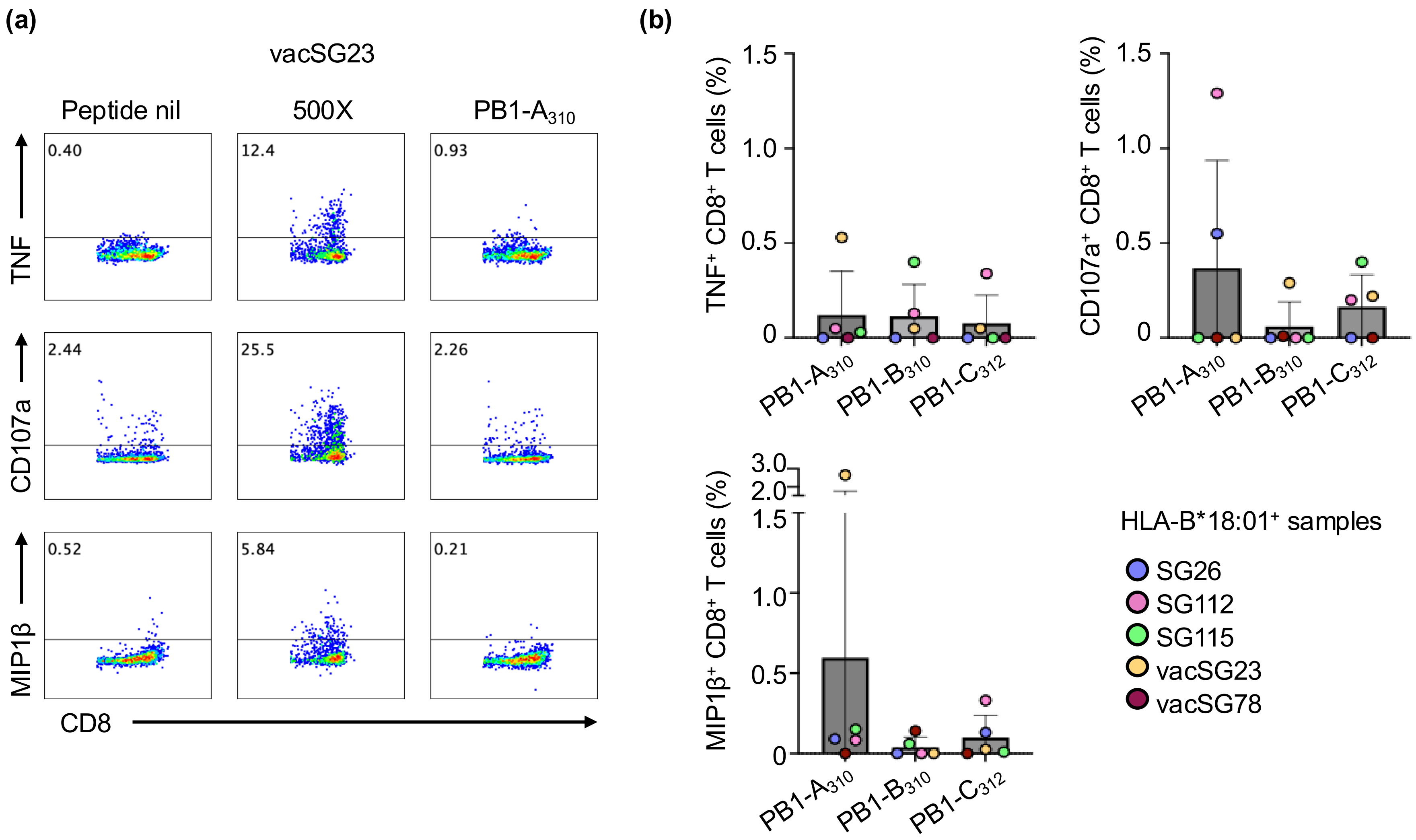

Supplementary Figure 7

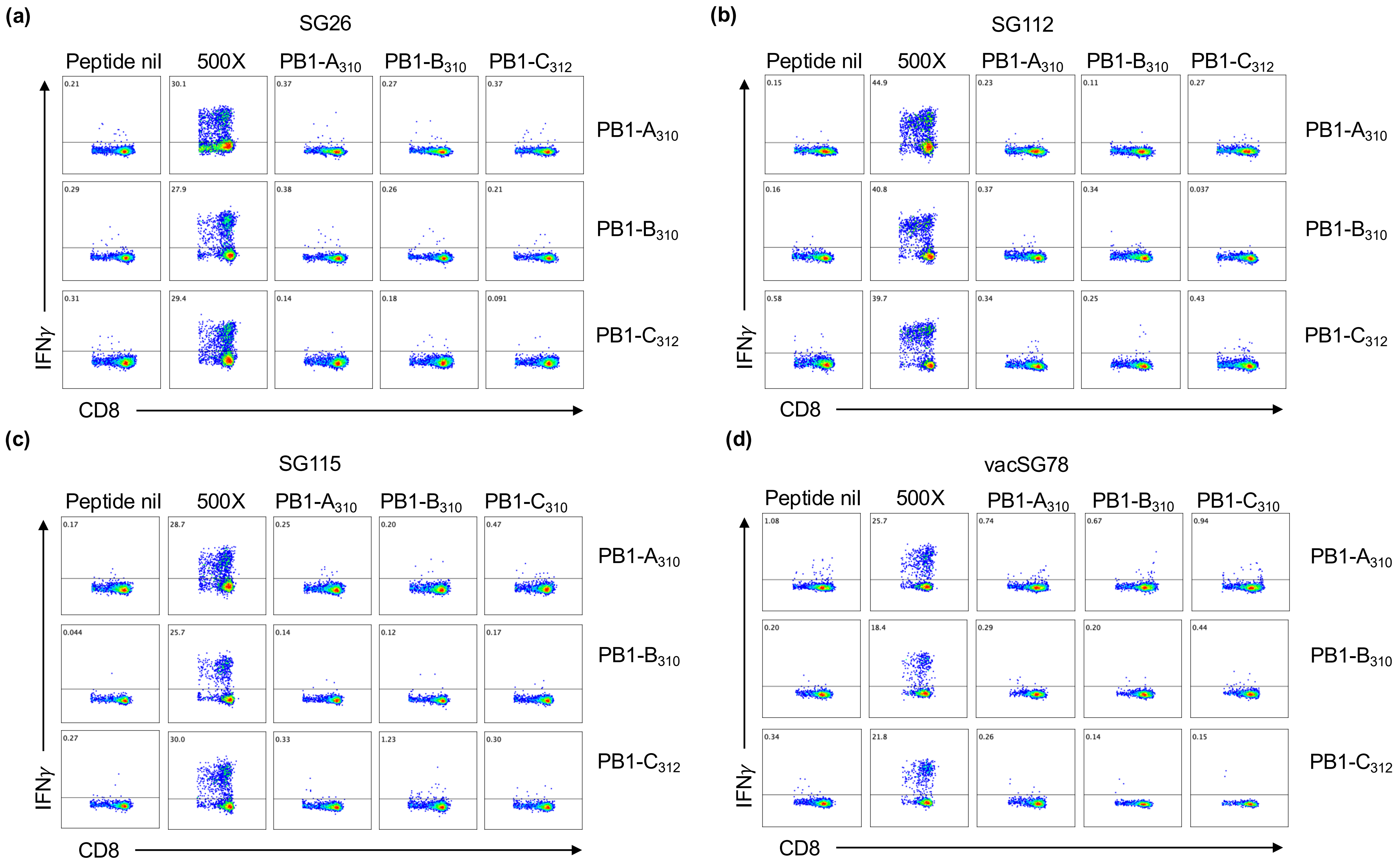

Supplement: Supplementary file 1 [file CTI2-15-e70123-s002.pdf]
