# Supplementary material for: Assessing CD8+ T cell cross‐reactivity to influenza A, B and C virus‐derived peptides presented by HLA‐B*18:01
Source: Clin Transl Immunology. 2026 Oct 13;15(10):e70123. doi: 10.1002/cti2.70123 (PMC13659276; doi:10.1002/cti2.70123)
Supplement: Supplementary file 2 [file CTI2-15-e70123-s001.docx]

**Supplementary Table 1. Crystallographic data collection and refinement statistics of HLA-B*18:01 presenting PB1-derived peptides**

| Data Collection Statistics | HLA-B*18:01 PB1-A_310_ | HLA-B*18:01 PB1-B_310_ | HLA-B*18:01 PB1-C_312_ |
| --- | --- | --- | --- |
| Space group | P 2_1_ 2_1_ 2_1_ | P 2_1_ 2_1_ 2_1_ | P 2_1_ 2_1_ 2_1_ |
| Cell Dimensions  (a, b, c) (Å) | 51.01, 81.73, 111.18 | 51.05, 81.95, 111.17 | 51.17, 81.43, 112.11 |
| Resolution (Å) | 46.36 – 1.80  (1.84 – 1.80) | 46.39 – 1.60  (1.63 – 1.60) | 46.55 – 1.35  (1.37 – 1.35) |
| Total number of observations | 596922 (33498) | 534829 (27020) | 1373046 (69529) |
| Nb of unique observations | 43948 (2577) | 62385 (3125) | 103551 (69529) |
| Multiplicity | 13.6 (13.0) | 8.6 (8.6) | 13.3 (13.7) |
| Completeness (%) | 100.0 (100.0) | 100.0 (100.0) | 100.0 (100.0) |
| I/σI | 18.4 (3.2) | 15.6 (2.4) | 20.2 (3.4) |
| R_pim_^a^ (%) | 2.9 (24.5) | 2.5 (32.0) | 2.6 (32.2) |
| CC_1/2_ (%) | 99.9 (88.8) | 99.9 (81.1) | 99.9 (97.3) |
| Refinement statistics |  |  |  |
| R_factor_^b^ (%) | 19.6 | 20.0 | 18.0 |
| R_free_^b^ (%) | 23.1 | 21.1 | 19.0 |
| R.m.s.d. deviations from ideality |  |  |  |
| Bond lengths (Å) | 0.007 | 0.007 | 0.006 |
| Bond angles (°) | 0.921 | 0.919 | 0.902 |
| Ramachandran plot (%) |  |  |  |
| Favored | 98.15 | 98.94 | 98.15 |
| Allowed | 1.85 | 1.06 | 1.85 |
| Disallowed | 0 | 0 | 0 |
| PDB code | 9ZZ7 | 9ZZ8 | 9ZZN |

Values in parentheses are for the highest resolution shell.

CC_1/2_: correlation coefficient.

^a^R_pim_ = Σhkl [1/(N-1)]1/2 Σi | Ihkl, i - < Ihkl > | / Σhkl < Ihkl >.

^b^R_factor_ = Σhkl | | Fo | - | Fc | | / Σhkl | Fo | for all data except ≈ 5% which were used for R_free_ calculation.

**Supplementary Table 2. List of HLA-B*18:01 contacts with PB1-derived peptides**

| Peptide | Peptide residue | HLA residue |
| --- | --- | --- |
| PB1-A_310_ | P1-Asn | Tyr7, Tyr59, Arg62, Asn63, Tyr159, Try167 |
|  | P2-Glu^N-Oe1^ | Tyr7, His9^Nε2^, Asp63^Oδ1^, Ile66, Tyr99, Tyr159 |
|  | P3-Asn^N^ | Tyr7^OH^, Ile66, Gln155, Tyr159 |
|  | P4-Gln | Arg62, Ile66 |
|  | P5-Asn | Gln155 |
|  | P6-Pro | Thr69, Asn70, Thr73 |
|  | P7-Arg^Nη1-Nη2^ | Thr73, Ser77, Arg97, Asp114^Oδ2^, Trp147, Val152, Leu156 |
|  | P8-Met^O^ | Thr73, Ser77, Trp147^Nε1^ |
|  | P9-Phe^N-OXT^ | Ser77, Asp80^Nδ2^, Tyr84, Leu95, Ser116, Tyr123, Thr143, Lys146^Nζ^, Trp147 |
| PB1-B_310_ | P1-Asn | Tyr7, Tyr59, Arg62, Asn63, Tyr159, Try167 |
|  | P2-Glu^N-Oe1^ | Tyr7, His9^Nε2^, Ser24, Asp63^Oδ1^, Ile66, Tyr99, Tyr159 |
|  | P3-Cys^N^ | Ile66, Tyr99^OH^, Tyr159 |
|  | P4-Leu | Arg62, Ile66 |
|  | P5-Asn | Gln155 |
|  | P6-Pro | Thr69, Asn70, Thr73 |
|  | P7-Arg^Nη1- Nη2^ | Thr73, Ser77, Arg97, Asp114^Oδ2^, Trp147, Val152, Leu156 |
|  | P8-Ile^O^ | Glu76, Ser77, Trp147^Nε1^ |
|  | P9-Phe^N-OXT^ | Ser77, Asp80^Nδ2^, Tyr84, Leu95, Ser116, Tyr123, Thr143, Lys146^Nζ^, Trp147 |
| PB1-C_312_ | P1-Asn^Oδ1^ | Tyr59, Arg62^Nη1^, Asn63, Tyr159, Try167 |
|  | P2-Glu^N-O1^ | Tyr7, His9^Nε2^, Ser24, Asp63^Oδ1^, Ile66, Tyr99, Tyr159 |
|  | P3-Cys^N^ | Ile66, Asn70, Arg97, Tyr99^OH^, Leu156, Tyr159 |
|  | P4-Gln^Oε1^ | Gln65^Nε1^, Ile66, Thr69, Asn70 |
|  | P5-Gln^N-Oε1^ | Asn70^Oδ1^, Thr73, Tyr74, Arg97^Nε^, Trp147 |
|  | P6-Pro | Gln155, Leu156 |
|  | P7-Glu | Thr73, Trp147, Ala150, Val152 |
|  | P8-Ala^O^ | Thr73, Glu76, Ser77, Trp147^Nε1^ |
|  | P9-Tyr^OH-N-OXT^ | Ser77, Asp80^Nδ2^, Leu95, Arg97, Ser116^Oγ^, Tyr123, Thr143, Lys146^Nζ^, Trp147 |

Hydrogen bond interaction defined with superscript and within ≤ 3.5Å, and Van der Waals interactions are within ≤ 4Å, determined in PyMoL.

**Supplementary Table 3. List of HLA-B*18:01^+^ donors**

| Donor ID | Sex | HLA-B1 | HLA-B2 | Influenza vaccination | Age (year) |
| --- | --- | --- | --- | --- | --- |
| SG26 | Male | 18:01 | 35:01 | yearly | 78 |
| SG112 | Female | 18:01 | 15:01 | not available | 43 |
| SG115 | Male | 18:01 | 44:03 | not available | 41 |
| vacSG23 | Female | 18:01 | 40:02 | yearly | 35 |
| vacSG78 | Female | 18:01 | 08:01 | yearly | 35 |

**SUPPLEMENTARY FIGURE LEGENDS**

**Supplementary Figure 1. Thermal stability of PB1-derived Influenza peptides presented by HLA-B*18:01**

Graph showing the DSF of purified protein samples at a concentration of 5μM with the normalized florescence intensity (%) plotted against temperature (°C). The melting curve is represented by a different dashed line, with HLA-B*18:01-PB1-A_310_ as light green, HLA-B*18:01-PB1-B_310_ as salmon and HLA-B*18:01-PB1-C_312_ as light blue.

**Supplementary Figure 2. Electron density for Influenza derived peptides presented by HLA-B*18:01**

Unbiased electron density (Fo-Fc, green mesh at 3σ) and final electron density (2Fo-Fc, blue mesh at 1σ) for PB1-A_310_ (green sticks) (**a, d**), PB1-B_310_ (salmon) (**b, e)** and PB1-C_312_ (light blue) (**c, f**) presented by HLA-B*18:01 (white cartoon).

**Supplementary Figure 3. Primary anchor residues and backbone characteristics of the three PB1-derived peptides bound to HLA-B*18:01**

(**a**) The primary anchor residues at P2 for PB1-A_310_ (green), PB1-B_310_ (salmon) and PB1-C_312_ (light blue) and (**b**, **c**) and P9 are represented in stick. The HLA is shown as white cartoon and hydrogen bonds are indicated by black dashed lines. All panels use the same colour coding as per panel (**a**). (**c**) Overlay of the three crystal structures with the peptide backbones in cartoon and anchor residues represented as sticks. (**d**) Overlay of crystal structures available in the PDB for peptide-HLA-B*18:01 complexes (coloured accordingly to the key, **Table 1**) with the three PB1-derived peptides structures presented in this study. (**e**) Overlay of the PB1-C_312_ (light blue) and the PB1_177-B_ peptide (pink).

**Supplementary Figure 4. HLA-B*18:01** peptide **binding motif**

The sequence logo represents the amino‑acid preferences at each position of 9‑mer peptides predicted to bind HLA‑B*18:01 with high affinity using the NetMHCpan 4.1 server  ^69^. Letter height reflects the relative contribution and frequency of each residue at a given position. Prominent anchor residues are visible at position 2 (P2) with an E, and the C‑terminal position (PΩ) with a Y/F/L/M consistent with the canonical binding requirements of HLA‑B*18:01. The colour represents the chemical nature of the residues, with red for acidic residue, green for polar residue, black for hydrophobic residue and blue for basic residue. Figure generated using Seq2Logo Adapted from  ^52^.

**Supplementary Figure 5. Flow cytometry gating strategy**

Gating strategy used for intracellular cytokine staining assay analysed via Flowjo 10.10.0. (**a**) FACS plots of SG26 NEC-C pulsed CD8^+^ T cell line showing the gating strategy used to obtain IFNγ^+^, TNF, MIP1β and CD107a of CD8^+^ T cells used for Figures 3, 4 and Supplemental Figures 6 and 7. Cells were gated on lymphocytes, singlets, Live CD3^mid-high^, CD8^mid-high^ T cells and IFNγ^+^ CD8^+^ T cells.

**Supplementary Figure 6. Additional functional markers of PB1-A_310_, PB1-B_310_ and PB1-C_312_ specific T cell lines determining immunogenicity.**

Additional cytokine profiles CD8^+^ T cell responses in Figure 3. PBMCs of HLA-B*18:01^+^ cells were stimulated with either PB1-A_310_, PB1-B_310_ or PB1-C_312_ at 10 µM for 10 days^+^ for T cell expansion (n = 5). After 10 days, an ICS assay was completed by restimulating each T cell line with the cognate peptide at 10µM. A positive control 500X condition and nil peptide negative control was implemented for each T cell line. (**a**) Representative FACS plots of CD8 vs TNF, CD107a and MIP1β for vacSG23 PB1-A_310_ pulsed CD8^+^ T cell lines are included. (**b**) Bar graphs displaying mean ± SD of TNF, CD107a or MIP1β CD8^+^ T cells detected for each peptide via flow cytometry and gated as per Supplementary Figure 5. Each coloured dot represents a donor’s T cell response, while the bars illustrate the mean for each peptide.

**Supplementary Figure 7. Summary of T cell reactivity and cross-reactivity to PB1-A_310_, PB1-B_310_ and PB1-C_312_** **measured by** **intracellular cytokine staining**

Summary of CD8^+^ T cell cross-reactivity for PB1-A_310_, PB1-B_310_ and PB1-C_312_ in Figure 4. PBMCs typed with HLA-B*18:01 was stimulated with 10 μM of peptide to generate PB1-A_310_, PB1-B_310_ and PB1-C_312_ pulsed T cell lines (n = 5). An intracellular cytokine assay was completed with the cognate peptide and the other two peptides for each T cell line. (**a-d**) Summary IFNγ vs CD8 flow cytometry plots for donor SG26, SG112, SG115 and vacSG78, respectively.
